# Comparative Outcomes Between Surgical and Conservative Management of Mallet Thumb: A Systematic Review and Pooled Analysis

**DOI:** 10.1177/15589447241291600

**Published:** 2024-11-13

**Authors:** Emily Hirslund, Chad Patience, Philip Hang, Armaghan Dabbagh, Mike Szekeres

**Affiliations:** 1 Faculty of Health Science, Western University, London, ON, Canada; 2 Faculty of Medicine, University of Toronto, ON, Canada

**Keywords:** distal phalanx, Zone T1, thumb, mallet thumb, surgery, hand therapy, function

## Abstract

**Background:**

While mallet finger remains a relatively common injury of the hand, mallet thumb is much rarer in occurrence. Mallet thumb management has been noted infrequently within the literature and reliable evidence regarding the most effective method of management remains absent. The aim of this review is to assess the quality of literature that exists pertaining to mallet thumb to determine whether conservative or surgical management is superior.

**Methods:**

A search was completed in February 2023 of Ovid Medline, Embase, CINAHL, and SPORTDiscus with no limitation on study type, and date of publication. Comparative outcomes of thumb interphalangeal (IP) joint range of motion, tip, lateral pinch and grip strength, complications, outcome measure scores, and follow-up period were recorded. We assessed 103 mallet thumbs (51 surgically and 52 conservatively managed) across the 23 studies of low to moderate quality based on the Structured Effectiveness Quality Evaluation Scale. The authors adhered to the Preferred Reporting Items for Systematic Reviews and Meta-Analyses guidelines.

**Results:**

While recommendations remain weak due to low quality of evidence, our review found a lower complication rate and higher IP joint flexion in thumbs managed conservatively.

**Conclusion:**

These findings demonstrate a need for future research to shift toward ensuring standardized patient-rated outcome measures are utilized and functional outcomes are included in research planning and operationalization in order to contextualize clinical outcomes.

## Introduction

A mallet thumb injury is an extensor tendon injury of the interphalangeal (IP) joint of the thumb that may or may not involve an avulsion injury to the distal phalanx.^1^ Mallet thumb injuries account for 2% to 3% of all mallet injuries.^2^ Literature observing the different types of management for this condition has been scarce and is often grouped with outcomes for treatment of mallet finger,^3-5^ making specific comparison of the management techniques for this unique condition challenging. Both conservative management including the use of various splints/immobilization and surgical management that employs various fixation methods have been reported to be effective in treatment of mallet thumb.^1,6-8^ In 2015, Abe et al^6^ completed a retrospective review and compared these outcomes to findings from a subsequent literature review. Their conclusion is that there is no significant difference between conservative and surgical treatment on extension lag, flexion/extension and time for immobilization, but surgery may lead to a more rapid recovery based on earlier mobilization with fixation. Since then, further case reports and case studies with regards to management of mallet thumb^9-11^ have been published to inform treatment of mallet thumb.

The aim of this study is to provide an updated systematic review on the current state of conservative versus surgical treatment of mallet thumb and provide insight with regards to treatment of mallet thumbs. We aim to assess the quality of literature that exists and use functional data to derive an answer to the above posed question.

## Materials and Methods

The authors adhered to the Preferred Reporting Items for Systematic Reviews and Meta-Analyses guidelines. This protocol was registered with Prospero with the registration number CRD42023420846, dated 06/06/2023.

### Data Identification

A search was completed in February 2023 of Ovid Medline, Embase, CINAHL, and SPORTDiscus with no limitation on study type, and date of publication. The keywords used in this search were: distal phalanx, terminal phalanx, base, Zone T1, Verdan, thumb injuries, mallet thumb, mallet hallux, extensor injury of the thumb, avulsion, intra-articular, fracture, distal interphalangeal joint, treatment or surgery or rehabilitation or occupational therapy or physiotherapy, orthosis, splints, therapeutics, range of motion (ROM), continuous passive, function, and rupture. The assistance of a Health Sciences librarian at Western University was used to ensure an efficient search strategy. Within this study, the term surgical is defined as a procedure that involves puncture below the dermis, including indirect and direct Kirschner wire (K-wire) fixation, screw fixation, plate fixation, and anchor fixation methods. Conservative management includes the use of various splints/immobilization and subsequent rehabilitation^6^ and is a procedure that does not involve puncture of the dermis.

### Eligibility Criteria

#### Design

Studies published in English were included within the study. There was no restriction on the date of publication. All study designs were included with the exception being for articles and educational material that does not state outcomes of treatment of intervention.

#### Participants

Adults (age > 18 years) with mallet thumb injuries that were treated with both surgical management as well as conservative interventions were included within the study. We did not include patients where the results of treatment for mallet thumb were not stated or could not be differentiated from the results for mallet finger.

#### Intervention/comparator

All surgical and conservative management protocols were accepted.

#### Outcomes

Thumb IP joint extension (initial/final), extensor lag, IP joint flexion (initial/final), tip pinch, lateral key, grip strength, complications, Visual Analog Scale (VAS) score, Disabilities of the Arm, Shoulder, and Hand (DASH) score, Quick Disabilities of the Arm, Shoulder, and Hand (Quick DASH) score, Patient-Rated Wrist Evaluation (PRWE) score, and follow-up period were identified as outcomes of interest within the studies.

#### Time

All isolated mallet thumb injuries regardless of chronicity.

### Study Selection

Three authors (EH, CP, PH) independently screened titles, abstracts, and full reviews for the studies included. Bibliography citation files were screened and reviewed by each author under the same criteria as were outlined for articles within Covidence. Each article required two votes to be moved on to the next review stage. All reviewers were blinded to the votes of others. Journal articles that had conflicts were resolved by a third reviewer within the research group. References were screened to identify any additional journal articles that matched the inclusion and exclusion criteria set out in the list of included studies.

### Data Extraction

Data were extracted by two independent reviewers. Any disagreements were flagged and further discussed with a third reviewer until a consensus was reached. A spreadsheet was used for organizing the data and the following data was extracted:

1. Studies: authors, country of origin, type of study, study location, conflict of interest, inclusion/exclusion criteria within the study, statistics tests used within study.
2. Participants: sample size, number of thumbs, gender, avulsion %, open/closed injury, surgical procedure, post-surgical rehabilitation protocol, conservative management protocol, radiological assessment, recovery time, follow-up time
3. Outcome measures and results: IP joint ROM at initial and final visit, extensor lag, tip pinch, lateral key pinch, grip strength, # of complications, types of complications, VAS score, DASH score, QuickDASH score, and PRWE score.

### Data Synthesis and Analysis

Extracted data on participants, surgical interventions, conservative interventions and outcomes were summarized using a predefined data extraction form including design, population, intervention and outcomes of interest. The studies were sorted to two groups: conservative and surgical management. Within this study, the term surgical is defined as a procedure that involves puncture below the dermis, including indirect and direct K-wire fixation, screw fixation, plate fixation and anchor fixation methods. Conservative management is defined as a procedure that does not involve puncture of the dermis, including immobilization as well as use of orthosis. The data was subsequently reviewed and analyzed within our research group. Six of the 23 studies reported extension and/or flexion values simply as “full.” To make use of these reports, we have assumed “full flexion” to mean at least 80 degrees and “full extension” to mean at least 0 degrees. These assumptions fall in line with The American Academy of Orthopedic Surgeons normative thumb IP joint extension and flexion.^12^ Within the 23 articles included in this study, diverse techniques for surgical and conservative management of mallet thumbs were used with a mix of different treatments and comparators. As a result of this and the small number of studies around the topic, gathering enough data could not be aggregated to perform a true meta-analysis. In light of this, we attempted a pooled analysis to be able to directly compare the two management strategies.

### Critical Appraisal and Quality Assessment

The Structured Effectiveness Quality Evaluation Scale (SEQES) tool was used to assess the quality of the selected studies.^13^ The SEQES is a 24-item critical appraisal tool that evaluates the methodological characteristics of the study in seven general sorts (study questions, design, subjects, interventions, outcomes, analysis, and recommendations). A quality score between 33 and 48 indicates high quality, scores between 17 and 32 indicate moderate quality, and ≤16 indicates low quality. Each article was reviewed by 2 independent reviewers and any conflicts were resolved by a third reviewer.

## Results

### Search and Selection of Studies

**Figure 1.**
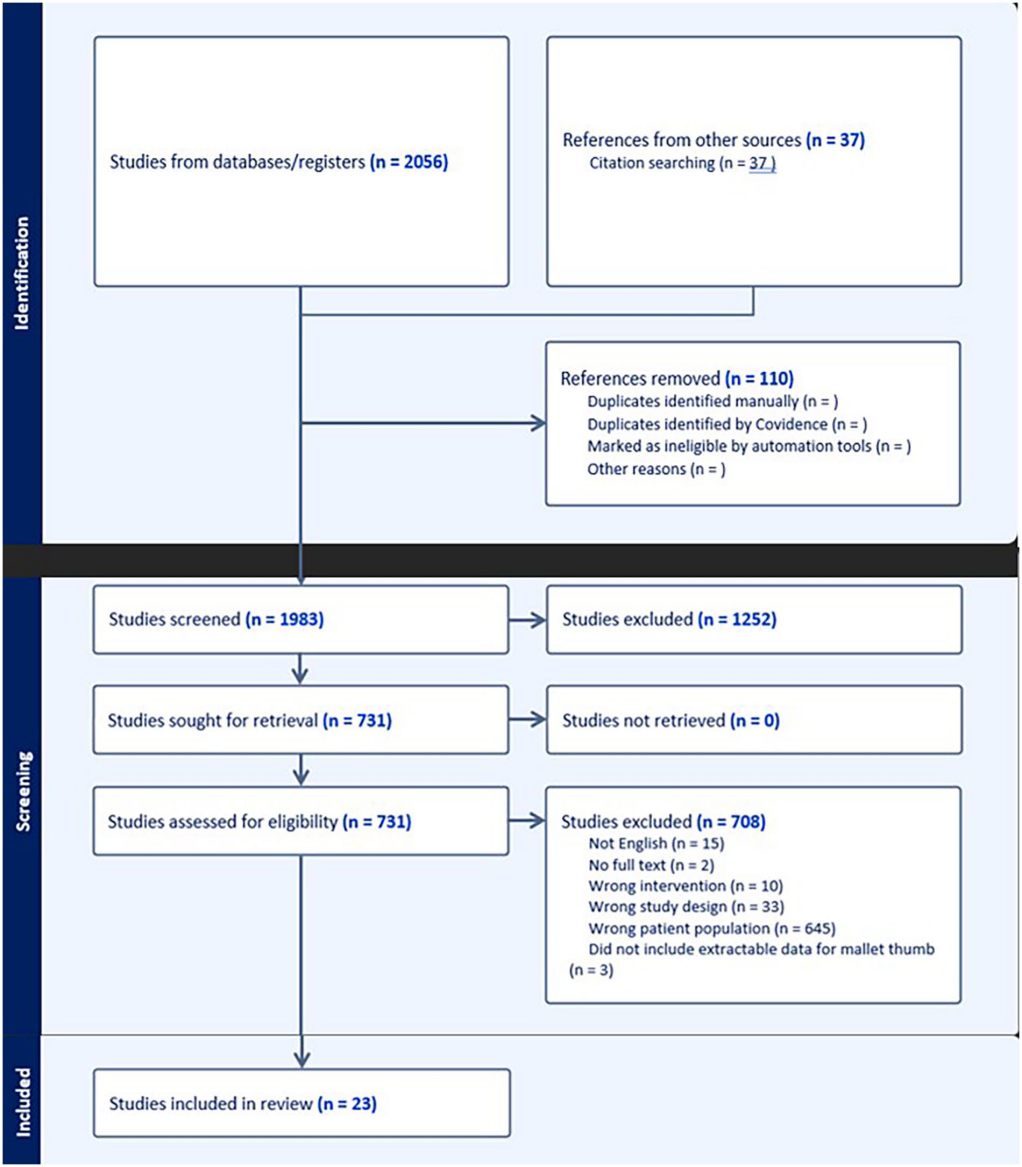
PRISMA flow diagram outlines the flow of study identification and selection.

Totally, 2056 studies were obtained from the four databases used. Out of which, 37 studies were identified from citation searches; 110 duplicate articles were removed, and the remaining 1983 articles were then screened. And 731 full-text studies were assessed for eligibility and after this review process, 23 studies were included in the systematic review (Figure 1).

### Quality of Studies

Structured Effectiveness Quality Evaluation Scale (SEQES) scores for the selected studies ranged from 6 to 27 out of a possible score of 48. The average score for all included studies was 15.5. The majority of studies (17 out of 23) were assessed to have low quality (Table 1). The remaining 6 studies were of moderate quality. The low SEQES scores are largely attributed to the studies lacking prospective data, no ability to randomize treatment or blind patients and assessors, and the lack of a control group. Thumb IP joint ROM, tip pinch, lateral pinch, grip strength, complications, outcome measures scores as well as follow-up period were outcomes of interest.

**Table 1.** SEQES Evaluation Scores for Quality of Research.

	1	2	3	4	5	6	7	8	9	10	11	12	13	14	15	16	17	18	19	20	21	22	23	24	Total
Aerts et al (2013)	1	0	1	1	0	1	1	0	1	0	0	2	2	0	0	1	0	1	0	0	0	0	2	2	**16**
Abe et al (2016)	1	1	1	1	0	1	1	0	1	2	0	2	1	1	2	1	1	1	1	0	2	1	2	2	**26**
Arvanitakis et al (2017)	1	0	1	2	0	1	1	0	1	0	0	2	1	0	0	1	0	1	0	0	0	0	1	2	**16**
Afshar et al (2021)	0	0	1	0	0	1	1	0	1	0	0	2	0	1	0	1	0	1	0	0	0	1	1	1	**11**
Casmus et al (2008)	1	0	1	0	0	1	1	0	1	0	0	2	1	1	0	1	0	0	0	0	0	1	0	1	**12**
De Smet et al (2003)	0	0	1	0	0	1	1	0	1	0	0	2	0	0	0	1	0	1	0	0	0	1	0	1	**10**
Din et al (1983)	0	0	1	0	0	1	1	0	1	0	0	2	0	0	0	1	1	1	0	0	0	1	0	2	**12**
Kang et al (2012)	2	0	1	1	0	1	1	0	1	1	0	2	1	0	0	1	0	1	0	0	0	1	1	0	**16**
Kasstenberger et al (2022)	2	2	0	0	1	1	0	2	2	1	1	1	0	0	2	2	1	1	1	0	2	1	1	1	**25**
Lee et al (2013)	0	2	2	2	0	2	2	0	2	0	0	2	0	0	0	0	0	0	2	0	1	1	1	2	**24**
Lu et al (2018)	2	0	1	1	0	1	1	0	1	0	0	2	2	0	0	2	2	2	0	0	0	1	1	1	**16**
McCarten et al (1986)	1	0	1	0	0	1	1	0	1	0	0	2	0	0	0	0	0	1	0	0	0	1	1	1	**13**
Mifune et al (2016)	1	0	0	0	0	1	1	0	1	0	0	2	1	0	0	1	0	1	0	0	0	1	0	1	**9**
Miura et al (1986)	2	0	0	0	0	1	1	0	1	0	0	2	1	0	2	1	1	1	0	0	0	1	2	1	**12**
Mukasa et al (2019)	1	1	1	1	0	1	1	0	1	0	0	2	1	0	0	1	0	1	2	0	1	1	1	2	**24**
Nelis et al (2008)	1	0	1	1	0	1	1	0	1	1	0	2	2	1	0	1	0	2	0	0	0	1	1	1	**16**
Norrie et al (2013)	2	0	1	0	0	1	1	0	1	0	0	2	1	1	0	1	0	1	0	0	0	1	1	1	**13**
Nishimura et al (2013)	2	0	0	0	0	1	1	0	1	0	0	2	0	1	0	1	0	0	0	0	0	1	0	1	19*
Ofazoglu et al (2017)	1	1	1	1	0	1	1	0	1	0	0	2	1	1	2	2	1	1	2	0	1	1	1	2	**24**
Patel et al (1986)	2	0	1	1	0	1	1	0	1	0	0	2	1	0	0	1	0	1	0	0	0	1	1	1	**16**
Primiano (1986)	1	0	1	0	0	1	1	0	1	0	0	2	1	0	0	1	0	1	0	0	0	1	1	1	**13**
Tabbal (2009)	2	0	0	0	0	1	1	0	1	0	0	2	0	0	0	0	0	0	0	0	0	1	0	1	**9**
Yamanaka and Sasaki (1999)	1	0	0	0	0	1	1	0	1	0	0	2	2	0	0	0	0	0	0	0	0	1	2	1	**12**

* *x* = SEQES questions, ascending order.

*y* = Study/year

1. *Was the relevant background work cited to establish a foundation for the research question?*

2. *Was a comparison group used?*

3. *Was patient status at more than one time point considered?*

4. *Was data collection performed prospectively?*

5. *Were patients randomized to groups?*

6. *Were patients blinded to the extent possible?*

7. *Were treatment providers blinded to the extent possible?*

8. *Was an independent evaluator used to administer outcome measures?*

9. *Did sampling procedures minimize sample/selection biases?*

10. *Were inclusion/exclusion criteria defined?*

11. *Was an appropriate enrollment obtained?*

12. *Was appropriate retention/follow-up obtained?*

13. *Was the intervention applied according to established principles?*

14. *Were biases due to the treatment provider minimized (ie, attention, training)?*

15. *Was the intervention compared with the appropriate comparator?*

16. *Was an appropriate primary outcome defined?*

17. *Were appropriate secondary outcomes considered?*

18. *Was an appropriate follow-up period incorporated?*

19. *Was an appropriate statistical test(s) performed to indicate differences related to the intervention?*

20. *Was it established that the study had significant power to identify treatment effects?*

21. *Was the size and significance of the effects reported?*

22. *Were missing data accounted for and considered in analyses?*

23. *Were clinical and practical significance considered in interpreting results?*

24. *Were the conclusions/clinical recommendations supported by the study objectives, analysis, and results?*

### Participant Demographics

A total of 103 participants across the 23 studies were evaluated, with 51 participants having their mallet thumb surgically managed and 52 managed conservatively (Table 2). Ninety-seven participants had their sex reported. Yamanaka and Sasaki^4^ and Lu et al^14^ identified the number of male and female participants, but studied both mallet fingers and mallet thumbs and did not report sex specific to type of injury. Nelis and Wouters^15^ did not identify the sex of any of their participants. Overall, 68% of participants were male. Ninety-one percent of male mallet thumbs in this review were closed injuries compared to 94% of female mallet thumbs. Twenty-five thumbs from Miura et al^16^ could not be used as the study did not individually report the sex of the participants and the type of mallet thumb they had. Ninety-one percent of surgically managed mallet thumbs and 58% of conservatively managed thumbs were closed injuries. Thirty-three of 51 (64.7%) surgically managed mallet thumbs involved avulsion fractures compared to 17 of 51 (33.3%) conservatively managed ones. The injuries were most often reported as hyperflexion, direct blow, or laceration injuries, but Oflazoglu et al^1^ and Kastenberger et al^9^ reported specific mechanisms of injury like “fall down the stairs,” “table saw injury,” or “hit by a soccer ball.”

**Table 2.** Study Characteristics.

Authors (year)	Study design	Patient count (thumbs included/total participants)	Sex	Injury mechanism
Aerts et al (2013)	Case report	1/1	F	Forced hyperflexion
Abe et al (2016)	Retrospective case series	10/10	*N* = 10 M	*N* = 9 hyperflexion, *n* = 1 direct blow
Arvanitakis et al (2017)	Case report	1/1	F	Hyper flexion
Afshar et al (2021)	Case Report	1/1	M	Fall
Casmus et al (2008)	Case report	1/1	M	Football
De Smet et al (2003)	Case report	1/1	M	Unknown
Din et al (1983)	Case report	4/4	*N* = 4 M	*N* = 1 kick, *n* = 1 crush, *n* = 1 hyperflexion, *n* = 1 laceration
Kang et al (2012)	Case series	1/16	M	Fall
Kasstenberger et al (2022)	Case series	16/16	*N* = 13 M, *N* = 3 F	*N* = 2 fall from bicycle, *n* = 3 slip and fall, *n* = 3 sporting, *n* = 3 heavy object fell on thumb, *n* = 2 motorcycle accident, *n* = 1 thumb caught, *n* = 2 could not recall
Lee et al (2013)	Case report	1/1	M	Crush
Lu et al (2018)	Case report	1/30	Unknown	Unknown
McCarten et al (1986)	Case report	2/2	*N* = 2 M	Hyper flexion
Mifune et al (2016)	Case report	1/1	F	Hyper flexion
Miura et al (1986)	Case series	25/25	*n* = 15 M *N* = 10 F	*N* = 15 laceration, *n* = 3 crush, *n* = 3 sprain, *n* = 1 contusion
Mukasa et al (2019)	Case report	1/1	M	Hyper flexion
Nelis et al (2008)	Case series	3/9	Unknown	*N* = 2 work-related, *n* = 1 sports
Norrie et al (2013)	Case report	1/1	F	Direct blow
Nishimura et al (2013)	Case report	1/1	M	Direct blow
Ofazoglu et al (2017)	Retrospective Study	22/416	*N* = 14 M *N* = 8 F	*N* = 6 fall, *n* = 2 unknown, *n* = 1 laceration, *n* = 3 basketball, *n* = 1 jammed thumb, *n* = 2 tennis, *n* = 1 injury playing with son, *n* = 1 flag football, *n* = 1 catching bottle, *n* = 1 suicide attempt, *n* = 1 ice hockey, *n* = 1 football, *n* = 1 box fell on thumb
Patel et al (1986)	Case series	2/2	*N* = 1 F *N* = 1 M	*N* = 1 direct blow, *n* = 1 fall while skiing
Primiano (1986)	Case series	2/2	*N* = 1 F *N* = 1	*N* = 1 direct blow, *n* = 1 fall while skiing
Tabbal (2009)	Case report	1/1	M	Hyper flexion
Yamanaka and Sasaki (1999)	Case report	2/16	Unknown	Unknown

### Surgical Procedures

A multitude of surgical techniques were used to treat the mallet thumbs in this study. Mallet thumbs that did not have an avulsion fracture were largely managed with tendon sutures both with and without Kirschner wire (K-wire) IP joint pinning. One of the 18 non-avulsed mallet thumbs from Oflazoglu et al^1^ was managed with K-wire alone but did not describe the technique used. Mallet thumb fractures were repaired through percutaneous pinning, internal fixation (ORIFs), or suture anchors. Most (94%) of the studies included the specific technique used for each repair. Avulsion fractures treated with percutaneous pinning included direct K-wire fixation with and without IP joint trans fixation, indirect K-wire fixation,^9^ fixation with biodegradable Meniscus Arrows^®^,^15^ and fixation with compression fixation pins.^5^ ORIFs were done with screws^1,17^ or a modified hook plate.^18^ Suture anchoring was done both with and without K-wire IP joint transfixation.^1,6,10,11,18-21^

### Conservative Management Techniques

In similarity to surgical management, there was a wide variance in the conservative management approaches of mallet thumbs. Immobilization methods consisted of the use of Stack, coil, or custom mallet splints; opponens splints; and short-term casting followed by Stack or custom mallet splinting. The initial, continuous thumb immobilization phase ranged from 2 to 8 weeks in duration, with 6 weeks being the most common.^1,7,8,11,16,22-24^ Two thumbs had their immobilization technique reported but did not have a prescribed time frame.^1^ Ten thumbs were reported as “continuous splinting” and a prescribed timeframe but did not outline the type of splint used.^1,24^ Five thumbs from Oflazoglu et al^1^ did not report any information on the type of treatment used. Twelve of 52 thumbs used night splinting of 2, 4, or 6 weeks.^1,7,8,22^ Miura et al^16^ had patients wear their coil 8 to 12 hours per day for 3 to 6 months after the initial 4 to 6 weeks of full-time use. No two studies used the exact same immobilization type and duration.

### Range of Motion

Seventeen of 23 studies included ranges of motion for both follow-up flexion and extension. From those 16, Abe et al,^6^ Arvanitakis et al,^10^ and McCarten et al^11^ also provided pre-treatment extension ROM, but no pre-treatment flexion values. Only Patel et al^8^ and Primiano^24^ included pre-treatment and follow-up values for both flexion and extension. Patel et al,^8^ however, did not report on pre-treatment flexion range of one of the 4 treated mallet thumbs. 4 studies^1,15,20,21^ did not report any ranges of motion for any points in time. Lu et al^14^ reported both pre-treatment and follow-up extension without any flexion values and Casmus and Burroughs^10^ reported follow-up extension ROM alone. All studies that reported ranges of motion presented them in degrees.

Average, resultant thumb IP joint flexion for the 39 reported thumbs in the surgical group was 58.4 degrees compared to 74.2 degrees of average flexion for 24 reported conservative thumbs. Resultant extension for the 40 surgically managed thumbs that were reported was +10.8 degrees and the same for that of the conservatively managed thumbs was +2.3 degrees. When comparing resultant ROM by sex, male participants had an average, resultant flexion of 63.2 degrees across 28 thumbs compared to 65 degrees from 6 thumbs of female participants. Average extension for males was +3.3 degrees from 29 thumbs versus +5.8 degrees across 6 thumbs in the reported female population.

### Strength

Measurements of strength were very limited across the included studies and the methods authors used to report them was very heterogeneous. Only Arvanitakis et al,^10^ Kastenberger et al,^9^ and Lu et al^14^ assessed any strength of tip pinch, lateral key pinch, or grip. Arvanitakis et al^10^ measured only lateral key pinch at follow-up. Kastenberger et al^9^ measured all three types of strength at follow-up and reported their averages: tip pinch 4.5 kg for 15 thumbs, lateral key pinch 6.4 kg for 15 thumbs, and grip strength 35.6 kg for 16 thumbs. Lu et al^14^ assessed tip and lateral key pinch at two separate follow-ups, but amalgamated both values at their respective time point and reported them as percentages of that on the opposite side (79% of opposite at 8 weeks and 91% at 12 weeks). No studies that conservatively managed mallet thumbs assessed grip or pinch strength at any point in time.

### Patient-Rated Outcome Measures

Abe et al^6^ and Kastenberger et al^9^ were the only included studies to assess patient-rated outcome measures (PROM). Both studies used the QuickDASH and Kastenberger et al^9^ also used the PRWE and Mayo Wrist Score (MWS). All outcome measures were taken on follow-up only and not pre-treatment. Twenty-four surgically managed mallet thumbs had at least one outcome measure taken. No conservatively managed mallet thumbs had a PROM assessed. The average QuickDASH score for the 24 assessed thumbs^6,9^ (all 16 thumbs from Kastenberger et al and 8 of 10 thumbs from Abe et al) was 9.4 and the average PRWE score from the 16 thumbs in Kastenberger et al^9^ was 11.9. The average MWS score in Kastenberger et al^9^ was 78.1.

### Complications

There were 9 thumbs with reported complications in the surgical group making for a complication rate of 17.6% across all 51 included thumbs. Two complications were from tendon sutures, 3 from screw fixations, and 4 from K-wire procedures. Both tendon suture repairs with complications also had K-wire IP joint fixations, meaning they could also be included in the K-wire complication grouping. The types of surgical complications were nail deformity (1 with tendon suture and 1 with K-wire fixation), infection (2 with K-wire fixation), wound necrosis (1 with screw fixation), extension deficit (1 with tendon suture), prolonged pain due to a tight cast (1 with screw fixation), prolonged nausea (1 with K-wire fixation), prominent hardware (1 with screw fixation), and pull-out wire breakage requiring open surgical removal (1 with K-wire fixation). One of the thumbs fixated with K-wire had both a nail deformity and infection. 6 of the surgical complications were in males, 2 in females, and in one case, sex was not reported.

The rate of complications with the conservatively managed thumbs was much lower compared to surgically managed mallet thumbs: there was one self-reported complication: a rate of 2.4%. Eleven thumbs from Miura et al^16^ could not be included in this value as they were secondarily excluded or lost to follow-up prior to completion of treatment. When considering reported complication rate based on injury type, closed mallet thumbs had a reported complication rate of 12% compared to a 4% rate in open mallet thumbs.

Since one would expect there to be more risk with having surgery and, thus, the potential for a higher complication rate, we decided to identify separate complications around ROM alone in hopes of creating a purer comparison of surgical and conservative management. This was also done due to ROM deficits being infrequently reported as a complication despite some thumbs having more restricted resultant range than those whose range was reported as a complication. To our knowledge, there are no established definitions of restricted thumb IP joint extension or flexion, so some were developed based on the studies reviewed. Abe et al^6^ reported one thumb to have the complication of “extension deficit.” Using this information, we have assumed an extension complication is a resultant thumb IP joint extension ROM of 10 or more degrees short of 0 degrees extension (ie, contracted into 10 or more degrees of flexion). Primiano reported a complication in one of the two thumbs treated where the resultant flexion ROM was 45 degrees and defined it as a “loss of flexion.”^23^ Because of this, we have assumed a flexion ROM complication to mean a resultant range of 45 degrees or less.

Using our definitions, 12 surgically managed thumbs (31%) had a flexion complication compared to 2 conservatively managed ones (8%). Extension complications arose 2 times in surgical management (5%) and 1 time in conservative management (4%). Eighty percent of ROM complications were male, but this comparison may be misleading, as 2 of 17 ROM complications (1 thumb from Lu et al^14^ and 1 thumb from Miura et al^16^) did not have their sex specifically identified and female participants only making up 32% of the total number of participants reviewed.

The ROM complication rate for closed mallet thumbs with reported ROM data was 30% with flexion complications 6 times more likely than extension ones. Identifying ROM complications for open injuries proved difficult. Twenty of 25 thumbs in Miura et al^17^ were open injuries. Fourteen of those 25 thumbs had their ROM reported but did not have their injury type identified. Additionally, 1 open mallet thumb in Oflazoglu et al^1^ did not have ROM values reported for use in ROM complication calculation. Because of this, only 5 open mallet thumbs could be considered, making for a ROM complication rate of 40% which is almost certainly inflated. All ROM complications that could be identified for open injuries were flexion related. Lastly, none of the thumbs in the included studies had both an extension and a flexion limitation after management.

## Discussion

This systematic review compares the outcomes following surgical and conservative management for mallet thumb injuries. There was a total of 103 participants across the 23 studies, 51 participants were managed surgically and 52 were managed conservatively. Average final thumb IP joint flexion was 58.4 degrees for the surgical group, and 74.2 degrees in the conservative group (Table 3). Average final thumb IP joint extension was +10.8 degrees in the surgical thumbs and +2.3 degrees in conservatively managed thumbs (Table 3). These data demonstrate that conservatively managed thumbs tended to achieve higher IP joint flexion than those managed surgically. Surgically managed thumbs achieved greater hyperextension than those managed conservatively.

**Table 3.** Results.

Authors (Year)	Management type	Final IP joint ROM (ext/flex)	Number of complications	Follow-up period (weeks)
Aerts et al (2013)	Conservative	0/30	None	14 weeks
Abe et al (2016)	Surgical	+5/40; 10/55, 0/60, +10/55, +10/55, +10/70, +10/60, +5/55, +10/60, +20/75	1	*n* = 1 32 weeks; *n* = 2 20 weeks; *n* = 2 16 weeks; *n* = 3 24 weeks, *n* = 1 0 weeks
Arvanitakis et al (2017)	Surgical	0/65	None	52 weeks
Afshar et al (2021)	Surgical	0/45	None	78 weeks
Casmus et al (2008)	Conservative	“full”/Unknown	None	Unknown
De Smet et al (2003)	Conservative	0/75	None	12 weeks
Din et al (1983)	Surgical	0/45, 0/25, 0/45, 0/45	None	*n* = 4 8weeks
Kang et al (2012)	Surgical	0/“full”	1	Unknown
Kasstenberger et al (2022)	Surgical	Range: 0-45 (STD 12) / 26-94 (STD 22).	5	*n* = 1 528 weeks, *n* = 1 336 weeks, *n* = 1 164 weeks, *n* = 1 144 weeks, n = 1 108 weeks, *n* = 1 672 weeks, *n* = 1 488 weeks, *n* = 1 68 weeks, *n* = 1 496 weeks, *n* = 1 444 weeks, *n* = 1 380 weeks, *n* = 1 696 weeks, *n* = 1 280 weeks
Lee et al (2013)	Surgical	Unknown	None	Unknown
Lu et al (2018)	Surgical	17/Unknown	1	Unknown
McCarten et al (1986)	*n* = 1 Surgical, *n* = 1 conservative	*n* = full/full (surgical), *n* = full	None	Unknown
Mifune et al (2016)	Surgical	5/80	None	16 weeks
Miura et al (1986)	Conservative	*n* = 9 “full” extension, *n* = 5 “not full,” 11 lost/excluded; *n* = 14 “no thumbs had limited flexion,” 11 lost/excluded	5	*N* = 25 24 weeks
Mukasa et al (2019)	Surgical	=8/90	None	24 weeks
Nelis et al (2008)	Surgical	Unknown	None	*n* = 3 6 weeks
Norrie et al (2013)	Conservative	“full”/“full”	None	16 weeks
Nishimura et al (2013)	Surgical	=16/94	None	28 weeks
Ofazoglu et al (2017)	*n* = 6 surgical, *n* = 16 conservative	*n* = 22 unknown	1	Surgical: *n* = 2 Unknown; *n* = 1 7.6 weeks, *n* = 1 3 weeks, *n* = 1 5 weeks, *n* = 1 9.1 weeks. Conservative: *n* = 4 Unknown, *n* = 1 5.3 weeks; *n* = 1 6.3 weeks, *n* = 1 3.1 weeks, *n* = 1 5 weeks, *n* = 1 23 weeks, *n* = 1 14.3 weeks, *n* = 2.7 weeks, *n* = 1 0.9 weeks, *n* = 1 1.7 weeks, *n* = 1 4.9 weeks, *n* = 1 12.4 weeks, *n* = 1 9.7 weeks
Patel et al (1986)	Conservative	5/55; ‘+5/85; +5/75; +15/60	None	*n* = 3 24 weeks; *n* = 1 52 weeks
Primiano (1986)	Conservative	+25/75; 0/45	1	*n* = 1 60 weeks, *n* = 1 52 weeks
Tabbal (2009)	Surgical	Unknown	None	6 weeks
Yamanaka and Sasaki (1999)	Surgical	0/50; +15/85	None	*n* = 2 12 weeks

*Note.* IP = interphalangeal; ROM = range of motion; STD = Standard Deviation.

From a functional perspective and the authors experience providing hand therapy, achieving a higher degree of IP flexion is more important than hyperextension for daily functional hand use, such as gripping and grasping tasks. Another interesting finding to highlight is the higher rate of complications observed in the surgically managed mallet thumbs. There was a total of 9 reported complications in the surgically managed group (17.6% of included thumbs) and 6 complications in the conservatively managed group (11.5% of included thumbs). One may argue that if there is a choice of management, based on the increased complication rate associated with surgical management alone, conservative management may be better. However, it is noteworthy to mention that those thumbs that are managed surgically may have experienced a much more severe injury to begin with and direct comparison may not be fair. Specifically, the indication for surgery usually included the involvement of greater than 25% of the articular surface or the presence of a joint subluxation.^9^ Although conservatively managed thumbs obtained higher overall flexion, the average flexion for surgically managed thumbs at 58.4 degrees is still within functional limits.

To further classify complications into a functional picture, we classified complications based on ROM complications as previously outlined. Using our definitions, 12 surgically managed thumbs (31%) had a flexion complication compared to 2 conservatively managed ones (8%). Extension complications arose 2 times in surgical management (5%) and 1 time in conservative management (4%). Again, surgically managed thumbs experienced a much higher rate of challenges in obtaining IP flexion, whereas extension complications were uncommon in both groups.

Our study found similar outcomes to the literature review by Kastenberger et al,^9^ demonstrating that both surgical and conservative approaches have a place in the treatment of mallet thumb with surgical management having a higher risk of complications. In contrast to Kastenberger et al,^9^ our review demonstrated that conservatively managed mallet thumbs achieved higher degrees of IP joint flexion (in conjunction with a reduced complication rate: 11.5%) and surgically managed thumbs achieved higher degrees of IP joint hyperextension (with an increased complication rate: 17.6%). These findings update the conversation around functional outcomes after management of mallet thumb. Though the recommendation may be weak due to low quality studies and a lack of data, our study indicates that conservative management may be more effective in maximizing a functional outcome while minimizing complications following mallet thumb injury. It is important to note however, that surgically managed thumbs still performed well overall and the final average flexion score was within functional limits for this group.

There were several challenges with analyzing the data within this study. Several studies grouped mallet thumb and its associated management with mallet finger data which made extracting data and associated comparisons challenging. There were numerous instances wherein there was no distinguishable data between these two injuries, despite there being a significant anatomical difference. All levels of injury were included in our study regardless of injury chronicity which can also impact outcomes, although rarely the status of chronicity was reported. Soft tissue mallets and bony mallets are included in this study, and their outcomes can also vary.There was a tendency within the surgical literature to disregard post-operative management details (such as splinting position, therapy considerations, movement considerations, and so on). Although it is essential to understand surgical technique, it is equally paramount to understand how these surgical techniques are protected post-operatively, and from a patient perspective—how therapists may best progress these repairs back into a functional digit for daily use and to understand the entire spectrum of management. Similar to surgical management, there was a clear variance in the approach to managing mallet thumbs conservatively. No two studies used the exact same immobilization type and duration. Future studies may look to compare immobilization protocols to determine a reproducible approach for future compactor studies to better utilize.

Abe et al^6^ and Kastenberger et al^9^ were the only included studies to assess PROM, indicating that most of the literature presented does not take into context the patient’s view of function or outcome. No conservatively managed mallet thumbs had a PROM assessed. These findings demonstrate a clear need for future research to shift toward ensuring standardized PROM are utilized and functional outcomes are included in research planning and operationalization in order to contextualize clinical outcomes.

Further limitations in this study included the inconsistent and unclear reports of assessment in ROM within included studies. Only 72% of included studies documented ranges of motion for both follow-up flexion and extension leading to needless challenges in intervention comparison. More than ¼ of included studies omitted final ROM data completely. Six of the 23 included studies reported extension and/or flexion values simply as “full” without prior determination of this term. Our study used the guidelines outlined by the American Academy of Orthopedic Surgery^7^ to determine the parameters of “full,” although it is highly likely that the various authors cited within our study have variable perspectives on what “full” entails. Future studies are encouraged to determine parameters for deeming ROM “full” and/or using a standardized approach to reporting ROM data, such as the American Academy of Orthopedic Surgery or the American Society of Hand Therapy.

## Conclusion

This study demonstrated that the evidence for the best treatment of mallet thumb is both scarce and weak, but supports both surgical and conservative types of management. Even though the complications rate were lower in the conservatively treated thumbs, no conclusive statements regarding the inferiority and superiority of surgical and conservative management of mallet thumb can be made. Future research should prioritize the inclusion of PROM in data extractions and inclusion of post-operative management processes. Specifically for therapists, it is recommended that a future study compares immobilization types and durations of immobilization for mallet thumb.
